# Injury and Illness Surveillance in Para-Cycling: A Single-Centre One-Season Prospective Longitudinal Study

**DOI:** 10.3390/sports13060158

**Published:** 2025-05-23

**Authors:** Thomas Fallon, Paul Carragher, Neil Heron

**Affiliations:** 1 Centre for Public Health, Queen’s University Belfast, Belfast BT7 1NN, UK; 2 Edinburgh Sports Medicine Research Network & UK Collaborating Centre on Injury and Illness Prevention in Sport (UKCCIIS), Institute for Sport, PE and Health Sciences, University of Edinburgh, Edinburgh EH8 9YL, UK; 3 Sport Ireland Institute, National Sports Campus, Abbotstown, 17301 Dublin, Ireland

**Keywords:** injury and illness surveillance, para-cycling, cycling, health

## Abstract

**Introduction:** Para-cycling is a competitive sport governed by the World Body for Cycling, Union Cycliste Internationale (UCI), encompassing various cycling disciplines tailored to athletes with physical or visual impairments. This study aimed to prospectively monitor the incidence of injury and illness in Para cyclists during the 2024 Paralympic season. **Methods:** This prospective, observational study included ten professional Para cyclists (five male, five female) with impairments ranging from spinal cord-related, neuromuscular, and musculoskeletal conditions to vision impairment. The definitions of an ‘athlete health problem’, ‘injury’, and ‘illnesses’ followed the Para sport translation of the IOC consensus. Injury and illness data were collected weekly using the Oslo Sports Trauma Research Centre Questionnaire on Health Problems V2 (OSTRC-H2), with the addition of subjective markers of well-being and training load, between February 2024 and October 2024. All medical contacts for any injury or illness were logged in line with consensus statement recommendations. **Results:** The OSTRC-H2 questionnaire had a response rate of 76.5% (±12.2%, range 55–88%) across the 35 weeks. Athletes reported 7.36 (95% CI: 5.41–9.46) health problems per 365 days, with a medical attention rate of 5.56 (95% CI: 3.91–7.36) per 365 days. The overall injury rate was 1.94 per 365 athlete days (95% CI: 1.23–2.93), with a higher incidence in males (2.44, 95% CI: 1.53–3.67) than in females (1.51, 95% CI: 0.68–2.95). Conversely, illness rates were higher in females (5.40, 95% CI: 3.00–8.11) than in males (1.80, 95% CI: 0.60–3.30), with an overall illness rate of 3.60 per 365 days (95% CI: 2.29–5.10). **Conclusions:** This is the first study to present prospective injury and illness epidemiology rates in Para cyclists in combination with subjective well-being markers. The findings underscore the importance and feasibility of longitudinal health monitoring in Para cyclists, ensuring that both physical and mental health concerns are systematically tracked and addressed. This enables a proactive, multidisciplinary support system to respond effectively to fluctuations in well-being, particularly during periods of injury or illness.

## 1. Introduction

Para-cycling is a competitive sport governed by the Union Cycliste Internationale (UCI), encompassing various cycling disciplines tailored to athletes with physical or visual impairments. Para-cycling can be broken down into two broad categories, Para Track and Para Road, with further subdisciplines arising from each [1]. Despite the sport’s growing competitiveness, there is a significant lack of epidemiological research on injuries and illnesses in Para-cycling, as well as in other cycling disciplines such as gravel, indoor, trial, and e-sport cycling (recent SR awaiting publication) [1]. Addressing this gap, prospective injury and illness surveillance has been identified as a key strategy for injury prevention [2]. Recognising this need, the UCI recently highlighted the importance of injury and illness epidemiology in their 2030 agenda in a drive to “*promote and support research in cycling epidemiology and medicine, especially for the benefit of lesser-known disciplines*” to inform evidence-based athlete health and safety strategies [3].

The foundational framework for injury prevention was introduced by Van Mechelen et al. in 1992, outlining a four-step model that begins with injury surveillance [4]. This model emphasises the necessity of monitoring injuries to establish a basis for effective preventive strategies. In 2006, Finch et al. expanded upon this initial model, introducing two additional steps to create the TRIPP (Translating Injury Prevention to Practice) framework [5]. This revised model underscores the importance of systematic injury and illness surveillance as a cornerstone for developing evidence-based prevention programs and for identifying the unique risk factors associated with injuries and illness within athletes.

Since 2021, cycling has had an International Olympic Committee (IOC) consensus statement [6] extension for the reporting of injuries in cycling [7]. There has been a Para sport translation of the IOC consensus statement for the reporting of injuries and illness within Para sports [8]. One notable difference between the cycling extension and the Para sports extension is the proposed expression of injury and illness rate in cycling. The IOC cycling extension suggests expressing injuries and illness per 1000 h, per 100 race days within Para-cycling, and per 365 days for comparison between disciplines. However, all studies that have been completed to date in Para sports have presented injury and illness incidence per 1000 athlete days. This challenges the comparisons that can be made on the injury and illness rates published between Olympic and Paralympic cycling disciplines and other Para sports. The IOC consensus statement and the Para sports translation both highlight the need for further prospective injury and illness monitoring in sports. This is to capture any health problem that an athlete has, not just those of medical attention health problems [6,8]. The Oslo Sports Trauma Research Centre Questionnaire on Health Problems (OSTRC) is a validated questionnaire [9,10] that has been used to obtain information on all health problems across a range of sports, including cycling and Para-cycling [11,12].

The International Paralympic Committee (IPC) has published injury and illness surveillance studies from each iteration of the Paralympic Games since London 2012. These studies suggest the rate of injury amongst all Para athletes ranges between 10 and 19 illnesses [13,14,15] and 10 and 27 injuries per 1000 athlete days [15,16,17,18]. Specifically, the rates of injury in Para-cycling at the Tokyo and Rio Paralympic games were 5.8 [15] and 7.0/1000 [16] per 1000 athlete days. Illness rates at the Rio Paralympic Games were 10.5/1000 athlete days [14] and at the Tokyo Paralympic Games were 4.7/1000 days [15]. There have been separate systematic reviews [19,20,21] and single-centre retrospective [22,23] and prospective [11,12,24,25,26,27,28] studies in Para sports injuries and illness. However, there remains limited research, particularly focused on Para-cycling, presenting injury and illness rates [12]. Therefore, it is difficult to ascertain the rate of injury or illness specifically within Para-cycling. A recent meta-analysis carried out as part of a multi-disciplinary systematic review on cycling injuries and illnesses found that, within Para-cycling, the injury rate per 365 athlete days is 3.85 (95% CI 3.59–4.10), and the illness rate is 3.89 (95% CI 3.42–4.36) per year [1]. Past systematic reviews carried out on mountain biking [29,30] and on road cycling [31] suggest the upper limb to be the most injured region and abrasions to be the most common injury type. Despite the recent increase in Para sports injury and illness surveillance [26,27,28,32,33], two studies have focused specifically on Para-cycling [12,23] that found the shoulder to be the most common injury location and respiratory illness to be the most common illness type.

Well-being is a “complex construct rooted in health, philosophy, and psychological practices” (p. 16) [34]. An athlete health problem is broadly defined as when an athlete “moves from *any* state of health to a ‘less-healthy’ state,” which undoubtedly affects one’s well-being [8]. Longitudinal monitoring of health problems within Para athletes has been conducted across various nations [11,22,26,32,33,35]. Recent research has shown the link between mental distress and injury and illness in Para athletes and the importance of longitudinal monitoring [25,33]. However, the combination of subjective self-report measures, which offer a practical, cost-effective approach to monitoring the effect of an athlete’s health problem, has been less commonly used. Therefore, further prospective research that integrates subjective measures of well-being with injury and illness surveillance is needed in Para-cycling to better understand both the types and effects of health problems, including their incidence, burden, and severity [12,36].

This study aimed to:

1. Conduct prospective surveillance of all self-reported and medical attention injuries and illnesses among professional Para cyclists using the OSTRC framework.
2. Evaluate how health problems affect subjective athlete-reported outcomes, including fatigue, stress, mood, sleep, and training volume/intensity.

We hypothesise that self-reported health problems in Para cyclists will be associated with significant changes in self-reported measures of fatigue, stress, mood, sleep quality, and training capacity. This study will provide medical professionals working within elite Para-cycling with an understanding of the injury and illness profiles seen within cycling and how they compare to other cycling disciplines.

## 2. Methods

### 2.1. Study Design and Participants

This was a prospective, longitudinal study that aimed to investigate injury and illness patterns among professional endurance Para cyclists. This study followed the injury reporting guidelines published in the Para sports translation of the IOC consensus on recording and reporting data for injury and illness in sports and the IOC consensus cycling-specific extension [6,7,8]. Ethical approval for this study was obtained from the Medical Faculty at Queen University Belfast, Northern Ireland (Faculty REC Reference Number: MHLS_23_175; date: 12 January 2024) and by the Sport Ireland Research Ethics Committee.

The participants were ten professional endurance Para cyclists, five males and five females, preparing for the 2024 Paris Summer Paralympic Games. A professional Para cyclist is defined as a cyclist who competes at the national/international level and receives a regular salary or income for their involvement in the sport [1]. The team medical staff (TF) of the National Paralympic Committee provided athletes with detailed information about the proposed study and obtained informed consent from all athletes.

### 2.2. Injury and Illness Definitions

In Para-cycling, “a health problem is when an athlete moves from *any* state of health to a ‘less-healthy’ state”, irrespective of its consequences on sports participation or performance or whether they have sought medical attention. This may include, but is not limited to, injury, illness, pain, or mental health conditions [8]. When athletes required medical attention, these contacts were divided into two separate definitions that align with the IOC consensus on recording and reporting data for injury and illness in sports [6,7].

1. Injuries were defined as “tissue damage or other derangement of normal physical function due to participation in sports, resulting from rapid or repetitive transfer of kinetic energy requiring medical attention”.
2. Illness was “a complaint or disorder experienced by an athlete, not related to injury. Illnesses include health-related problems in physical (e.g., influenza), mental (e.g., depression) or social well-being, or removal or loss of vital elements (e.g., air, water, warmth) requiring medical attention.”

### 2.3. Survey Design and Data Collection

The Oslo Sports Trauma Research Centre Questionnaire on Health Problems (OSTRC-H2) was used to capture athlete wellness [9,10]. The questionnaire has high validity and has reliability been used in recent Para athlete research, with consistently high compliance [9,10,26,27,28]. The OSTRC-H2 consists of four graded key questions about sports participation, training volume, performance, and health problems experienced during the previous 7 days. Question 1 (participation) was the ‘gatekeeper’ question; if athletes reported ‘full participation without health problems’, all further questions were not asked. Other subjective questions around health and well-being, rating fatigue, mood, stress, sleep, travel (most common mode of travel > 90 min), and diet were recorded weekly. All data were collected using an online Google Form, which aligned with the templates proposed in the recent Para Consensus.

The survey was piloted by doctors, physiotherapists, physiologists, and Para cyclists to ensure the survey was accessible and fit for purpose. During the pilot testing, it was suggested that the self-reporting section for symptoms be excluded from the athlete’s log due to its length and its impact on the accessibility features, specifically the voiceover function, which athletes with visual impairment rely on. The voiceover feature, which reads aloud the text on the screen, became overwhelming because of the large amount of text in the self-report section.

As a result of this feedback, it was agreed that athletes would be asked a simpler question: “Do you have a new health concern that you need medical attention for?” In addition, a free-text section was provided for athletes to report any symptoms. The revised approach was reviewed weekly by the lead physiologist. Weekly updates were provided to the wider support team using the Goalscape platform, with follow-up as deemed appropriate by support personnel [37].

All medical professionals logged medical contacts through a separate arm of the same survey. The survey and reminders were applied to each athlete’s online training calendar using the online software Training Peaks V12.74.0 (Training Peaks^®^, Boulder, CO, USA). Every Sunday, the system generated an email, and push notifications were delivered to the athletes’ smartphones via Training Peaks^®^. A reminder was sent out 2 days later each week by the same automated means.

### 2.4. Descriptive and Statistical Analysis

All weekly data were downloaded into an EXCEL database (Microsoft Excel 2018, Windows) and anonymised with a Personal Identification Number (PIN). Training metrics were downloaded for each session at the end of the study period from Training Peaks (Training Peaks^®^, Boulder, CO, USA). The metrics, training time (Hrs), energy spent (KJ), distance (KM), and training stress score (TSS) were taken from this data, which allowed injury and illness incidence rates to be accurately expressed across all exposure metrics. This enabled the accurate representation of injuries per 1000 h and per 365 athlete days. Competition periods were calculated from Day 1 (Opening) of the competition to the closing of the competition.

All data were processed on a Macintosh computer using Microsoft Office and R statistics (version 4.1.1). The methods applied included frequencies (%), crosstabs, and descriptive statistics. Multiple imputation was performed at the individual level for missing data [38]. A two-sample Poisson test was used to compare rates of injury and illness between different cycling demographics, including sex, impairment, injury location, and injury/illness type. Multivariable Poisson regression models were used to assess the impact of participation status (full participation vs. health problem) on mood, stress, fatigue, and sleep quality. Models were adjusted for training intensity and then training volume to isolate the influence of participation health status on the reported factors. The injury and Illness risk ratio (RR) was calculated and is presented with 95% confidence intervals (CIs) using R statistics (version 4.1.1; R Core Team). All the cyclists were analysed together, and the different cycling demographics (sex, impairment, injury location, and injury/illness type) were analysed separately to compare injury and illness data. All the statistical tests were two-sided, and results with *p* < 0.05 were statistically significant.

## 3. Results

A total of ten elite Para-cycling endurance athletes participated in this prospective injury and illness surveillance project, conducted over 35 weeks between February and October of the 2024 Paralympic season. The athletes had a range of impairments, including neurological spinal cord-related disorders, neuromuscular disorders, musculoskeletal impairments, and vision impairment (Table 1). Throughout the study, the athletes competed for a total of 410 competition days across the categories C division, Women, Men Blind Tandem, and Hand Cycling.

During the monitoring period, a total of 2430 training days were accumulated, leading to 4168.32 (±85.44) hours, 89,348.73 (±3057.59) kilometres ridden, and 2,066,677.03 (±69,956.13) kilojoules of energy expended. On average, each athlete totalled 416.8 (±85.4) hours, 8934 (±3057) kilometres, and 206,667.70 (±69,956.1) kilojoules.

**Figure 1:**
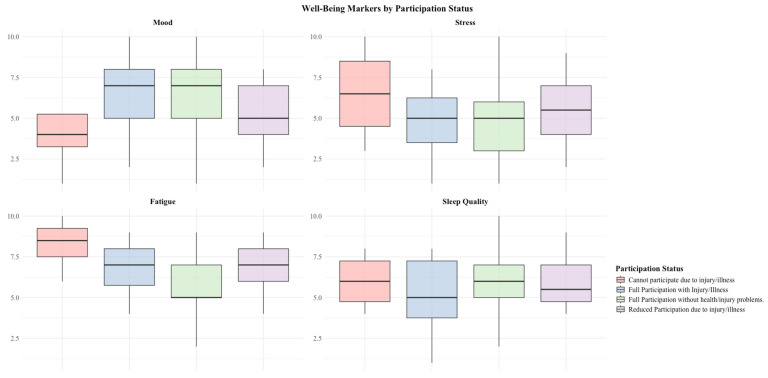
Well-being markers based on OSTRC-H2 gatekeeper questions.

**Figure 2:**
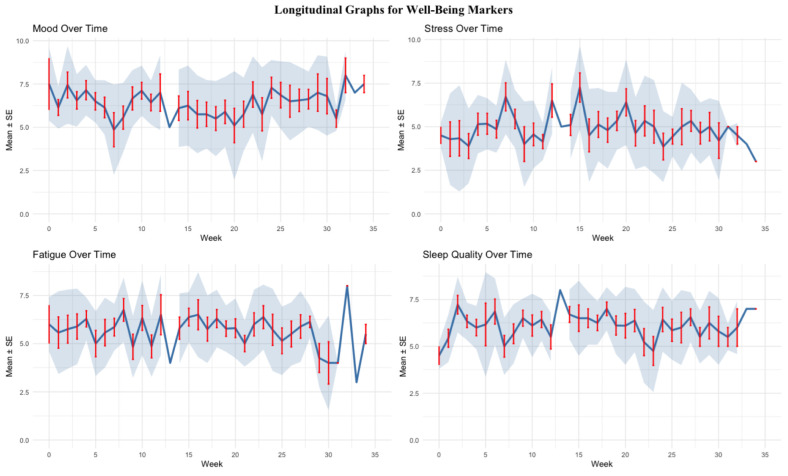
Longitudinal well-being markers.

The OSTRC-H2 questionnaire had a response rate of 76.5% (±12.2%, range 55–88%) across the season. The observed prevalence of an athlete’s health problem was 15.3%. Through using the questionnaire, athletes reported 7.36 (95% CI: 5.41–9.46) health problems per 365 days, with a medical attention rate of 5.56 (95% CI: 3.91–7.36) per 365 days (Figure 1). A two-sample Poisson test found no significant difference between the reporting rate of health problems through the OSTRC-H2 and the medical log, with a reporting rate ratio of 1.32 (95% CI 0.84, 2.08, *p* = 0.235). Multivariable Poisson regression models adjusting for training volume and intensity found that athletes reporting a health problem also reported significantly higher levels of perceived fatigue (*p* < 0.001), while no significant associations were found with mood (*p* = 0.088), sleep (*p* = 0.134), or stress (*p* = 0.223) (Figure 1 and Figure 2).

Injuries were primarily reported during the competition (46.2%, CI: 19.2–74.9%), followed by training (30.8%, CI: 9.1–61.4%) and a portion categorised as unknown (23.1%, CI: 5.0–53.8%) due to gradual onset. There was no injury reported to be associated with the athlete’s impairment. A total of 107 days were lost to injury during the monitoring period. The overall injury rate across both sexes was 1.94 (95% CI: 1.23–2.93), while the total illness rate was 3.60 (95% CI: 2.29–5.10) per 365 athlete days. The injury risk ratio (RR) (females vs. males) was 0.62 (0.19–1.93), and the illness RR (females vs. males) was 3.00 (0.91–13.52). The competition injury incidence per 365 days was 53.41 injuries (95% CI: 10.67 to 96.16), with a training injury incidence per 365 days of 9.03 injuries (95% CI: 1.12 to 16.95).

Males had a higher injury rate, while females reported a greater incidence of illnesses (Table 2). Males reported eight injuries and six illnesses, resulting in an injury rate of 2.44 (95% CI: 1.53–3.67) per 365 athlete days and an illness rate of 1.80 (95% CI: 0.60–3.30) per 365 athlete days. The average injury severity for males was 10.2 days (95% CI: 7.40–13.0), with the overall injury burden being 7.66 days per 365 days (95% CI: 5.57–9.75) and 12.2 days per 1000 h (95% CI: 9.34–15.06). In contrast, females reported five injuries but a significantly higher number of 18 illnesses, yielding an injury rate of 1.51 (95% CI: 0.68–2.95) and an illness rate of 5.40 (95% CI: 3.00–8.11) per 365 athlete days. The average severity of illness for females was higher at 2.94 days (95% CI: 2.15–3.73), with the illness burden being 2.65 days per 365 days (95% CI: 2.28–3.02) and 12.7 days per 1000 h (95% CI: 10.5–14.9).

Males have a higher injury rate per 1000 h (3.72, 95% CI: 1.39–6.51) compared to females (2.48, 95% CI: 0.50–4.96). Conversely, females exhibited a notably higher illness rate per 1000 h (7.44, 95% CI: 3.97–11.41) than males (3.25, 95% CI: 0.93–6.04). The injury rates per energy expenditure (kJ), TSS, and distance covered showed similar patterns, whilst females consistently experienced higher illness rates across these measures.

### 3.1. Types and Locations of Injuries

A total of 13 injuries were reported, with injuries occurring primarily during competitions (46.2%, 95% CI: 19.2–74.9%) and training (30.8%, 95% CI: 9.1–61.4%) (Table 3). One injury without a specified region is not included in Table 3. Injury rates were the highest in the shoulder region, with a rate of 0.60 per 365 days (95% CI: 0.15–1.20) and 0.90 per 1000 h (95% CI: 0.23–1.81). Cartilage injuries had the highest incidence at 0.45 per 365 days (95% CI: 0.00–1.05), equalling 0.68 (95% CI: 0.00–1.58) per 1000 h.

Head injuries were associated with the greatest injury severity, resulting in a loss of 13.67 days [95% CI: 9.48- 17.85] per injury, while fractures were the injury type with the highest injury severity, with 21.00 days [95% CI: 14.65–27.35] lost per injury. The location with the highest injury burden was the head, with 9.84 days lost [95% CI: 8.10–11.58] per 1000 h, whereas fractures represented the injury type with the greatest burden, accounting for 10.80 days lost [95% CI: 9.25–12.36] per 1000 h (Table 2).

### 3.2. Illness

The overall illness rate was 3.60 per 365 athlete days (95% CI: 2.29–5.10), with upper respiratory tract infections and gastrointestinal illnesses being the most reported. A total of 70 days were lost to illness over the monitoring period (Table 4). There was one ophthalmological illness reported, which the athlete was predisposed to by because of a pre-existing impairment. Illness occurred most frequently within 14 days following a camp or competition (56.52% [95% CI: 34.49%–76.81%]), followed by during a camp or competition (26.09% [95% CI: 10.23%–48.41%]) and outside these periods (17.39% [95% CI: 4.95%–38.78%]). The most common mode of transport used by athletes was air travel (77.0% [95% CI: 66.8%–85.4%]), followed by car (19.5% [95% CI: 11.8%–29.4%]) and train (3.45% [95% CI: 0.72%–9.75%]).

Respiratory illnesses were the most prevalent (56.0%), with a rate of 2.10 per 365 days (95% CI: 1.05–3.30), translating to 3.17 illnesses per 1000 h (95% CI: 1.58–4.98) (Table 3). All incidences of thermoregulatory illness occurred in visually impaired cyclists. Respiratory illness rates were significantly higher in females (1.65 per 365 athlete days, 95% CI: 0.75–2.70) compared to males (0.45 per 365 athlete days, 95% CI: 0.012–1.05). The incidence RR for respiratory illnesses between sexes was 3.66 (95% CI 0.71–6.61 *p* ≤ 0.05), indicating that female athletes were over three times more likely to experience respiratory illnesses than their male counterparts. Athletes with visual impairments exhibited a higher incidence of respiratory illnesses (1.20 per 365 athlete days, 95% CI: 0.369–2.0343) compared to those without impairments (0.60 per 365 athlete days, 95% CI: 0.012–1.1896). The incidence RR for respiratory illnesses in visually impaired riders was 1.66 (95% CI 0.58–4.75 *p* = 0.1125), suggesting a notable risk increase whilst not significant.

## 4. Discussion

This was the first study to present injuries and illnesses in professional Para cyclists in combination with subjective responses prospectively. Ten elite Para cyclists participated in a longitudinal health monitoring program. They were followed for 35 consecutive weeks while preparing for and competing at the world track championships in Rio de Janeiro, the Paris 2024 Paralympic Games, and the Road World Championships in Zurich. The main findings of this study are:

1. The overall injury rate was 1.94 (95% CI: 1.23–2.93) per 365 athlete days, while the overall illness rate was 3.60 (95% CI: 2.29–5.10) per 365 athlete days.
2. Males had a higher injury rate (2.44, 95% CI: 1.53–3.67) compared to females (1.51, 95% CI: 0.68–2.95), with an injury risk ratio (females vs. males) of 0.62 (95% CI: 0.19–1.93).
3. Females reported a significantly higher number of illnesses compared to males, resulting in an illness rate of 5.40 (95% CI: 3.00–8.11) per 365 athlete days for females and 1.80 (95% CI: 0.60–3.30) for males, with an illness risk ratio (females vs. males) of 3.66 (95% CI 0.71–6.61, *p* ≤ 0.05).
4. The shoulder region had the highest injury rate of 0.60 per 365 days (95% CI: 0.15–1.20).
5. Respiratory illnesses had a significantly higher incidence in females (1.65 per 365 athlete days, 95% CI: 0.75–2.70) compared to males (0.45 per 365 athlete days, 95% CI: 0.012–1.05), resulting in an incidence rate ratio (IRR) of 3.66 (95% CI: 0.71–6.61, *p* < 0.05).

### 4.1. Injury Rates

The injury pattern seen within this study is in line with previous findings seen within Para sports. The overall OSTRC-H2 response rate of 76.52% is comparable to the 75% reported in Norwegian Olympic Athletes [39] and 78.4% in Brazilian Para sport Athletes [28]. However, the findings are lower than the response rate seen within previous Para sports studies of 92.5 ± 8.5% [11]. We found that Para cyclists report 7.36 (95% CI: 5.41–9.46) health problems per 365 days, with a medical attention rate of 5.56 (95% CI: 3.91–7.36) per 365 days. This is similar to the 7.5 cases per athlete per year seen in Norwegian Para athletes [32] and to what has been seen in previous Para sports studies [36]. However, acknowledgement must be given to the difference in study design and the reporting rates per 1000 athlete days vs. the 365 athlete days completed within this study [20,22,24,28]. We present injury and illness incidences per 365 days and 1000 h in line with the IOC consensus extension recommendations for Para-cycling and previous road cycling consensus statements [7,40]. Our injury findings are comparable to what has been seen among German Para cyclists in terms of injury prevalence [12]. However, the incidence rates were not presented in that study, which limits our comparisons [12]. The injury incidence rate of 3.12 (95% CI: 1.44–5.04) per 1000 h is lower than what has been seen in Para-cycling at the Tokyo and Rio Paralympic games of 5.8 [15] and 7.0/1000 [16] per 1000 athlete days. Indeed, our findings are lower than the 3.85 (95% CI: 3.59–4.10) injuries per 365 days seen in the recent multidiscipline SR [1]. However, the injury rates are similar to what have been seen in other cycling disciplines, such as MTB Enduro 3.6/1000 h [41], road cycling 2.82/1000 h [42], and 2.52/1000 h [43], and lower than what have been seen at the Tokyo Olympic games at 1.5/1000 h [44]. Studies in road cycling report injury rates of 15.4 per 1000 h and BMX racing of 27.8/1000 h [44], which are much higher than the 3.12 (95% CI: 1.44–5.04) 1000 h reported in this study [45]. Comparisons between Paralympic Games injury rates and those from prospective cohort studies are limited due to differences in exposure days and event type/demands. In major competitions, exposure is concentrated over a short period of high-intensity effort, whereas, in a longitudinal study, training days are more frequent, which will be reflected in the exposure denominator. Para cyclists often have impairments that may limit their ability to train outdoors or train for extended periods, and thus, the absolute training volume may not appear as high as able-bodied peers. To validate this point, we presented injury exposures per 1000 h and 365 days in line with the IOC cycling extension recommendations for comparison between disciplines. We also presented overall injury and illness rates per 1000 km, 1000 TSS, and 1000 kilo Joules, which all show low overall exposures to injury and illness per 1000 kilo Joules, kilometres, and TSS [7,45]. In Para-cycling endurance disciplines, there are fewer specific specialist riders, and thus, there is a large crossover in riders who compete on the track and the road. Track races are much shorter and often more intense than road races. Thus, representing incidence rates per 1000 h for Para athletes and comparing with able-bodied disciplines may lead to an under-expression of the true incidence rate, and expressing illness per 365 athlete days is more reflective of the true injury exposure. Our study supports the recommendations of the IOC cycling consensus statement in presenting injury and illness rates in endurance Para-cycling disciplines per 1000 h and 365 days [7].

### 4.2. Subjective Markers

Para cyclists who reported a health problem reported significantly higher ratings of perceived fatigue and a trend of higher levels of stress during this period. Additionally, there was a notable trend of lower levels of mood and training volume and intensity during periods of illness and injury (Figure 1). Self-reported metrics, in addition to the OSTRC-H2 survey, can be an efficient way to monitor the effects of health problems on an athlete’s overall well-being [9,10]. Para athletes have been found to exhibit higher levels of mental distress during Paralympic Games [33]. Our findings suggest that an athlete’s health problem may be a determining factor in high levels of mental distress. This is supported by a recent study on Swedish elite Para athletes, which has found associations between reporting mental distress and experiencing an injury or illness affecting athletes’ participation in sports [25]. The addition of subjective well-being metrics can facilitate the application of a biopsychosocial and interdisciplinary approach in supporting Para sports athletes during this time. The addition of the Patient Health Questionnaire-4 (PHQ-4) may be an appropriate way of identifying athletes at risk of mental health problems in need of support [46,47]. Further prospective research is warranted in examining the effects of health problems on Para cyclists, especially on levels of mood, stress, and fatigue, given the higher rates of mental health problems seen in elite Para athletes [25,47].

### 4.3. Injury Locations and Types

The location and type of injuries across the year did differ. The most common location of injury is the shoulder, which is like what has been noted in past studies examining injuries in Para-cycling [12] and able-bodied cycling [29,30,31]. Injuries in cycling disciplines differ significantly from those in field sports and track and field [48,49,50]. While field sports and athletics tend to show a higher incidence of lower limb injuries, cycling primarily results in upper limb injuries. The most common injury type was cartilage injuries, which is slightly different to what has been seen in past cycling studies, with the most common injury type being abrasions/lacerations [31]. However, when Para cyclists crash, they may sustain a greater impact due to the inability to protect themselves from falling or even due to the location of injuries being exposed. There were no injuries that were pre-exposed due to the athlete’s impairment (i.e., rotator cuff shoulder pain in wheelchair athletes). This differs from what has been seen within previous Para-cycling studies, where there was a higher proportion of hand cyclists within the study sample, and thus, the prevalence of upper limb strains may be influenced by this fact [12]. Worth noting was the high injury burden seen with concussions and fractures. The incidence of concussions seen within this study was like that seen within a 52-week multisport study on Swedish elite Para athletes of 0.5/1000 h (95% CI 0.3–0.9). Para-cycling does not have a specific concussion diagnostic framework, and our findings show that concussions do happen in Para-cycling and carry the second greatest injury severity at 20.50 (95% CI 14.23, 26.77) days lost per injury. This has been a topic of recent debate, with calls for action around specific diagnostic frameworks for concussions in Para-cycling [51], with the first position statement of concussion in Para sports published in 2021 [52].

### 4.4. Illness

Our illness rates of 3.60 (95% CI: 2.29–5.10) per 365 days are like what has been reported in the recent systematic review of 3.89 (95% CI: 3.42–4.36) and what has been reported in previous Para-cycling studies [11,12,13,14]. However, they are similar to those reported at the Rio Paralympic Games of 10.5/1000 athlete days [14], although they are lower at the Tokyo Paralympic Games at 4.7/1000 days, which took place during COVID-19 with no spectators [15]. Illness surveillance during competition periods may underestimate the true incidence rate of illness due to the incubation periods often required for illness before symptom onset. Para cyclists have a higher rate of illness compared to injury, with respiratory illness having the greatest burden per year at 6.46 (95% CI: 2.51, 4.65) and gastrointestinal illness having the greatest severity at 4.00 (95% CI: 1.23, 6.77) days lost per illness. Over 56.52% (95% CI: 34.49–76.81%) of all illnesses occurred within 14 days of a training camp or competition. These findings differ from what was seen at the Tokyo 2020 Paralympics [15] and 2023 Parapan American Games [53], where the illness rates were at their highest in the pre-opening ceremony period. High rates of respiratory illness have been seen in previous Para sports studies. However, this is the first study to focus on Para-cycling [11,20,22,24,28,53,54,55]. Immunosuppression occurs after periods of acute higher training load, which may explain some of the patterns seen [56]. Our findings show that female athletes are at a significantly higher risk of respiratory illness, and visually impaired athletes also have higher rates of respiratory illness. This agrees with recent findings published from the Para Pan American Games [53]. These athletes and support staff (athlete assistants) should be particularly educated about hand hygiene, the use of disinfectants, mask-wearing, and avoiding hand shaking, especially with other teammates and staff, media, and social distancing (buffet environments, planes, and buses). Furthermore, athletes should consider supplementation such as zinc, vitamin D, and probiotics, which may help boost the immune system and reduce susceptibility [55,57]. We found that the most common mode of transport used by athletes was by plane (77.0%, CI: 66.8–85.4%). Consideration should be given to the increased risk of pathogens on flights during travel. Mitigation measures such as mask-wearing, awareness of higher-risk seating positions on aircraft, and limitation of movement around the cabin during flight should be given to all athletes before travel, with an additional emphasis on female and visually impaired athletes [57]. These findings are in line with recent IOC statements showing the risk of respiratory illness across all athletes and the need for more research to document the specific subgroups at higher risk [57,58].

Our study found three cases of thermoregulatory illness occurring in athletes with visual impairment. Visually impaired riders, known as stokers, will compete on the back of a tandem, tucked behind an able-bodied pilot. As the margins within Para-cycling tighten, aerodynamics has taken a big step forward, and because of reducing the overall aerodynamic drag of the tandem bicycle, the ambient cooling from wind flow is less for the stoker behind [59]. Our findings suggest that this may be a predisposing factor to thermoregulatory illness, such as exertional heat exhaustion. Medical staff and event organisers working within Para-cycling should consider adequate warm-up cooling strategies (ice vests, ice slush drinks, and fans); during the event (ice sponges, cold water bottles, ice socks, and misters); and post-event immediate cooling strategies (hand immersion, ice vests, and cool drinks) and a period of close monitoring during cooling down [60]. The same should be considered for Para cyclists with spinal cord injuries or neurological impairments, which impact the sweat rate and thermoregulation [60].

### 4.5. Limitations

This study contained a small elite sample size of equal gender balance between men and women. This limits the statistical power, increases the likelihood of Type II errors, and means the results may not reflect the wider population of Para cyclists. Consequently, the influence of impairment type, or classification, could not be explored in detail, despite likely contributing to the variations in health outcomes. As not every competition classification nor every para impairment was included in this study sample, this limits the generalisability of the findings within Para-cycling. The findings are exploratory, and the wide confidence intervals reflect a high degree of uncertainty. Further research with larger sample sizes is necessary to validate these findings and establish more robust conclusions.

### 4.6. Clinical Recommendations

These findings underscore the importance and feasibility of longitudinal health monitoring in Para cyclists, ensuring that both physical and mental health concerns are systematically tracked and addressed. This enables a proactive, multidisciplinary support system to respond effectively to fluctuations in well-being, particularly during periods of injury or illness, which may underpin psychological distress.

Athletes and support staff (athlete assistants) should be particularly educated about hand hygiene, the use of disinfectants, mask-wearing, and avoiding hand shaking, especially with other teammates and staff, media, and social distancing (buffet environments, planes, and bus). Furthermore, athletes should consider supplementation such as zinc, vitamin D, and probiotics, which may help boost the immune system and reduce susceptibility [55,57]. Mitigation measures such as mask-wearing, awareness of higher-risk seating positions on aircraft, and limitation of movement around the cabin during flight should be given to all athletes before travel, with an additional emphasis on female and visually impaired athletes.

Heat acclimatisation and pre/post-event cooling are warranted for all Para-cycling athletes, especially those with neurological conditions that impact the sweat rate/thermoregulation. Additionally, athletes with visual impairments would benefit from heat acclimatisation and pre/post-event cooling due to the lack of ambient cooling often linked with optimised positional extremes behind tandem pilots.

## 5. Conclusions

This is the first study to report injuries and illnesses among Para cyclists, including subjective wellness monitoring data. The findings indicate that the OSTRC-H2 Survey is a viable method for obtaining self-reported eHealth data from Para Cyclists with varying impairments. This study demonstrates the impact of athlete-reported health issues on overall well-being, with athletes experiencing higher levels of fatigue and stress, along with lower mood, during periods of injury or illness.

Whilst this study provides a preliminary insight into the health and well-being of elite Para cyclists, highlighting the potential and benefits of longitudinal health monitoring protocols to inform training changes and improve overall well-being. These findings should be interpreted with caution due to the small, heterogeneous sample and reliance on self-reported data. Despite these limitations, the study provides a foundation for future research and practical applications, encouraging a more personalised and proactive approach to athlete health management in Para sports settings.

## Figures and Tables

**Table 1:** Athlete classification category and impairment type (male/female number in parentheses).

Equipment Classification Category	Impairment Types	*n* (M/F)
**Bicycle (C division, C1–C5)**	Impaired passive range of motion	1 (1/-)
	Hypertonia/ataxia/athetosis	2 (1/1)
**Handcycle (H division, H1–H5)**	Impaired muscle power	1 (1/-)
**Tandem Bicycle (B division)**	-	6 (2/4)

**Table 2:** Overall injury and illness rates per 365 days and per 1000 h by sex (95% confidence interval (95% CI) in parentheses).

Sex	Total Injuries	Total Illnesses	Athlete Days	Injury Rate 365 (95% CI)	Illness Rate 365 (95% CI)	Injury Rate 1000 h (95% CI)	Illness Rate 1000 h (95% CI)	Injury Severity (95% CI)	Injury Burden per 365 Days (95% CI)	Injury Burden per 1000 h (95% CI)	Illness Severity (95% CI)	Illness Burden per 365 Days (95% CI)	Illness Burden per 1000 h (95% CI)
Male	8	6	1215	2.44 (1.53–3.67)	1.80 (0.60, 3.30)	3.72 (1.39–6.51)	3.25 (0.93–6.04)	10.2 (7.40,13.0)	7.66 (5.57–9.75)	12.2 (9.34–15.06)	2.43 (1.27, 3.59)	0.85 (0.61–1.09)	4.08 (2.82–5.34)
Female	5	18	1215	1.51 (0.68–2.95)	5.407 (3.00, 8.11)	2.48 (0.50–4.96)	7.44 (3.97–11.41)	14.2 (10.6, 17.8)	8.56 (6.30–10.82)	13.7 (10.5–16.9)	2.94 (2.15, 3.73)	2.65 (2.28–3.02)	12.7 (10.5–14.9)
Total	13	24	2430	1.94 (1.23–2.93)	3.60 (2.29–5.10)	3.12 (1.44–5.04)	5.28 (3.12–7.68)	12.0 (9.74,14.26)	16.2 (13.18–19.22)	25.9 (21.25–30.55)	2.80 (2.14, 3.46)	3.50 (2.94–4.06)	16.8 (14.2–19.4)

**Table 3:** Injury proportions, burden, and severity by location and type (number (n) and 95% confidence interval [95% CI] in parentheses).

Location	Concussion/Brain Injury (n)	Cartilage Injury (n) [95% CI]	Fracture (n) [95% CI]	Abrasion (n) [95% CI]	Joint Sprain (n) [95% CI]	Muscle Injury (n) [95% CI]	Bone Contusion (n) [95% CI]	Burden per 1000 h [95% CI]	Severity [95% CI]
**Shoulder**	-	-	1000% (2)	-	-	50% (1)	100% (1)	10.80 [9.25–12.36]	11.25 [9.48– 13.02]
**Knee**	-	66.7% (2)	-	-	100% (1)	-	-	2.40 [1.00–3.80]	5.00 [2.50– 7.50]
**Head, eyes, ears, teeth**	100% (2)	-	-	-	-	-	-	9.84 [8.10–11.58]	20.50 [14.23–26.77]
**Hand**	-	33% (1)	-	-	-	-	-	0.00 [0.00–0.00]	0.00 [0.00–0.00]
**Elbow and forearm**	-	-	-	100% (1)	-	-	-	0.48 [0.15–0.81]	2.00 [0.00–4.00]
**Lower Lumbar Region**	-	-	-	-	-	50% (1)	-	2.40 [1.00–3.80]	10.00 [3.80–16.20]
**Burden** **Per 1000 h**	9.84 [6.83–12.85]	1.68 [0.22–2.92]	10.08 [7.03–13.12]	0.48 [0.19–1.14]	0.72 [0.09–1.53]	2.40 [0.91–3.89]	1.98 [0.22–3.32]		
**Severity**	20.50 [14.23–26.77]	3.50 [0.91–6.09]	21.00 [14.65–27.35]	2.00 [0.77–4.77]	1.50 [0.200 3.20]	5.00 [1.90–8.10]	3.80 [0.91–6.59]		

**Table 4:** Illness rates, burden, and severity by system (number (n) and 95% confidence interval [95% CI] in parentheses) of the top 4 illnesses.

System	Percentage (n)	Rate per 365 Days [95% CI]	Burden per 365 Days [95% CI]	Severity per Injury [95% CI]
** Respiratory**	56.0% (14)	2.10 [1.05–3.30]	6.46 [2.51–4.65]	3.58 [2.51–4.65]
** Thermoregulatory**	12.0% (3)	0.45 [0.00–1.05]	0.30 [−0.26–1.59]	0.67 [−0.26–1.59]
** Gastrointestinal**	8.0% (2)	0.30 [0.00–0.75]	1.92 [0.29–1.62]	4.00 [1.23–6.77]
** Ophthalmological**	8.0% (2)	0.30 [0.00–0.75]	0.48 [−0.09–0.57]	1.00 [−0.39–2.39]

## Data Availability

The original contributions presented in the study are included in the article; further inquiries can be directed to the corresponding author.
